# Associations between the Healthy Eating Index-2015 and S-Klotho plasma levels: A cross-sectional analysis in middle-to-older aged adults

**DOI:** 10.3389/fnut.2022.904745

**Published:** 2023-01-11

**Authors:** Teng-Chi Ma, Jing Zhou, Chen-Xi Wang, Zhi-Zhi Lin, Feng Gao

**Affiliations:** Department of Cardiology, The Affiliated Hospital of Yan’an University, Yan’an, Shaanxi, China

**Keywords:** HEI, dietary pattern, Klotho, biomarker of aging, NHANES

## Abstract

**Background and aim:**

The Healthy Eating Index (HEI) is a dietary index developed by the United States Department of Agriculture (USDA) to determine whether a diet adheres to US dietary guidelines. Soluble Klotho (S-Klotho) is a protein with essential anti-aging properties. However, whether HEI is linked to S-Klotho plasma levels is still debatable. This study aimed to assess the association between HEI-2015 and S-Klotho in middle-to-older aged adults in the National Health and Nutrition Examination Survey (NHANES) from 2007 to 2016.

**Methods:**

The study included 8456 middle-to-older aged (40–79 years old) participants. Multivariate regression models were used to estimate the correlation between HEI-2015 and S-Klotho concentrations. General additive models and two-piece-wise regression models were used to investigate the possible non-linear relationships between HEI-2015 and S-Klotho concentrations. Moreover, a stratified analysis of potential influencing factors was performed.

**Results:**

A positive correlation was observed between HEI-2015 and S-Klotho plasma levels (β = 0.74, 95% CI: 0.21, 1.27, *P* = 0.0067). According to the two-piece-wise regression, the turning point of HEI-2015 was 45.15. When the range of HEI-2015 was from 0 to 45.15, the relationship between HEI and S-Klotho was insignificant (β = −0.87, 95% CI: −2.47, 0.73, *P* = 0.2858). However, when the range of HEI-2015 was from 45.15 to 100, HEI-2015 increased by 1 unit, the S-Klotho increased by 1.30 pg/ml (β = 1.30, 95% CI: 0.55, 2.05, *P* = 0.0007), suggesting a dose-response relationship. Furthermore, the stratified analysis showed that the association between HEI-2015 and S-Klotho concentrations was more significant in people with normal body mass index (P-interaction = 0.0161).

**Conclusion:**

There is a dose-response relationship between the HEI-2015 and S-Klotho in the middle-to-older aged adults. This relationship suggests that adherence to healthy dietary patterns may benefit the prevention of aging and health maintenance. The underlying mechanisms require further investigation.

## 1. Introduction

Dietary patterns are significant in nutritional epidemiology for characterizing and explaining dietary behavior. With the continuous development of methods for evaluating dietary patterns, it is now regarded as the basis of dietary guidelines (1). Epidemiological statistics data indicate an association between good dietary patterns and a reduction in the prevalence of various metabolic diseases and an increase in quality of life (2–4). The Healthy Eating Index (HEI) was originally designed by the United States Department of Agriculture (USDA) in accordance with the food pyramid and dietary guidance (5). The primary goal was to comprehensively evaluate and monitor the dietary status of American residents and to integrate whether the national diet fits the requirements of dietary guidelines and nutrients into a single index to accurately reflect dietary quality. The most recent version of indicators that can comprehensively and effectively assess whether the composition and content of daily food intake of residents are consistent with dietary guidelines was developed in 2015 (6).

In 1997, the Klotho gene was accidentally discovered and considered a gene related to aging, with decreased gene expression associated with a shorter life span (7). This protein was named after the Greek goddess “Clotho” for its molecular role in regulating metabolism and longevity. The expression product of the Klotho gene, the Klotho protein, is mainly found in the kidney, brain, and parathyroid glands (8–10). Klotho protein exists in two functionally different forms: a transmembrane form and a soluble form. Proteolysis on the membrane produces the stripped version of Klotho protein (S-Klotho). S-Klotho was identified as a circulating component detectable in serum, with direct effects on tissues and cells that do not produce Klotho, and is thought to have essential anti-aging properties (11). High levels of S-Klotho have been demonstrated to have protective effects on the heart, kidneys, bones, and cognition (12–15). Furthermore, the emerging evidence for the role of S-Klotho in health and disease has greatly contributed to our current understanding of the aging process, prompting researchers to investigate the underlying mechanisms and influences.

The regulatory role of diet in preventing and controlling aging is generating increasing interest. However, no research has been conducted to date that examines the association between HEI-2015 and S-Klotho. A clear understanding of whether healthy eating patterns can modulate S-Klotho appears crucial. Therefore, this study examined the relationship between HEI-2015 and S-Klotho in middle-aged to older people using data from the National Health and Nutrition Examination Survey.

## 2. Materials and approaches

### 2.1. Participants

The National Center for Health Statistics (NCHS) conducted a hierarchical, multi-phase study of the civilian non-institutionalized population in the United States and the District of Columbia. NHANES aims to assess health and nutritional status (16). Data were collected through interviews, physical examinations, and laboratory tests. The Centers for Disease Control (CDC) and the Ethics Review Committee of NCHS reviewed and approved all NHANES from 2007 to 2016. For the purposes of this research, data on NHANES from 2007-2008, 2009-2010, 2011-2012, 2013-2014, and 2015-2016 were searched. A total of 8,456 people aged 40–79 years old with completed 24-h dietary recalls and S-Klotho levels were included in the study, and participants with Metabolic Syndrome (MetS) as defined by the National Cholesterol Education Program’s Adult Treatment Panel III report (NCEP/ATPIII) (17) criteria were excluded. The study followed the Strengthening the Reporting of Observational Studies in Epidemiology-Nutritional Epidemiology (STROBE-NUT) guidelines (18).

### 2.2. Calculation of the HEI-2015

Data from the 24-h dietary recall of each person’s first day was utilized to construct the HEI-2015 for each individual. HEI-2015, derived from the USDA Food Model, is based on compliance with the dietary quality indicators of the 2015 US Diet guidelines. HEI-2015 consists of 13 ingredients, including ten food groups(Total Fruits, Whole Fruits, Total Vegetables, Greens and Beans, Whole Grains, Dairy, Total Protein Foods, Seafood and Plant Proteins, and Fatty Acids) and four moderation components (Refined Grains, Sodium, Added Sugars, and Saturated Fats). To obtain each person’s HEI-2015 score, we first summarized the amount of all dietary components associated with 13 HEI-2015 components, as well as the energy corresponding to the same dietary composition. Furthermore, the aggregate of these values was used to calculate 13 ratios for each individual, which were then scored according to the HEI score criteria for each component. Then the component scores are added together to calculate the total HEI-2015 score of each individual. The HEI total score ranges from 0 to 100. The better the diet, the higher the score. According to the USDA classification recommendation, we set the cutoff points as 60, 70, and 80 (6). An HEI total score of >80 is rated as “good diet,” a score of 60–80 is rated as “needs improvement,” and scores <60 indicate a “poor” diet.

### 2.3. S-Klotho plasma levels

Blood samples were collected from NHANES program participants between the ages of 40 and 79 years. All samples were stored at −80°C until they were scanned. The data were compared to those received on the electronic manifest and entered into the laboratory information system (19). For quality assurance, the average of two replicate analyses was employed, and samples with more than 10% replicate results were reanalyzed. S-Klotho was developed based on the solid-phase sandwich enzyme-linked immunosorbent assay kit (IBL International, Japan) (20).

### 2.4. Assessment of covariates

Potential confounding variables included the following:

The survey also collected demographic and lifestyle data, including age, gender, race, income, education, smoking status, alcohol consumption status, physical activity level, body mass index (BMI), systolic blood pressure (SBP), and diastolic blood pressure (DBP).

In addition, race/ethnicity was categorized as Mexican, other Hispanic, non-Hispanic white, non-Hispanic black, or other race; based on the family poverty-to-income ratio was categorized as low, moderate, and high income (PIR, <1.3, 1.3–3.49, ≥3.50); education level was categorized as less than high school, high school or equivalent, college or above; exercise status was categorized as <600, 600–1200, >1200; smoking status was categorized as daily, sometimes, and not at all, drinking status was categorized as never drinking (<12 drinks in lifetime), history of drinking (≥12 drinks in a year and no drinks in the last year, or no drinks in the last year but lifetime consumption of ≥12 drinks), and light drinkers (past year ≤ 1 drink per day on average for women or ≤2 drinks per day for men), moderate drinkers (≤3 drinks per day on average for women or ≤4 drinks per day for men in the past year), or heavy drinkers (≥4 drinks per day on average for women or ≥5 drinks per day for men in the past year).

At baseline, Glycated hemoglobin (%), glucose (mg/dL), cholesterol (mg/dL), HDL-cholesterol (mg/dL), LDL-cholesterol (mg/dL), triglycerides (mg/dL), uric acid (mg/dL), and C-reactive protein (CRP, mg/dL) levels were measured at the baseline. Furthermore, participants’ renal function was assessed by measuring eGFR using the executive summary of the KDIGO 2021 Clinical Practice Guideline (21).

### 2.5. Statistical analysis

The data in this study were statistically analyzed in accordance with CDC guidelines (22). This is because NHANES is designed to generate data representative of the non-institutionalized civilian population of the United States. For categorical variables, the chi-square test was used, and for continuous variables, a linear regression model was used to get the *P*-values. To test the correlation between HEI-2015 and S-Klotho plasma levels, a simple linear regression model was constructed: crude model, with no adjustment for age, sex, or race; model I, with adjustment for age, sex, and race; and model II, with adjustment for age, gender, race/ethnicity, education level, family poverty income ratio, physical activity, energy intake, chronic kidney disease, smoking and drinking habits. In order to investigate the possibility of non-linear correlation, the generalized additive model and spline smoothing were generalized. Additionally, the inflection point is determined by using two-piece-wise regression model. Finally, stratified analyses were performed according to sex, age (<60 years or ≥60 years), race/ethnicity (white or non-white), chronic kidney disease, body mass index (<25.00 or ≥25.00), and smoking and drinking habits. The statistical packages R (The R Foundation; version 4.2.0)^1^ and EmpowerStats (X&Y Solutions Inc.)^2^ were adopted to analyze data *P* < 0.05 was considered statistically significant.

## 3. Results

The current study included 8,456 participants (mean age: 57.38 ± 10.95; 60.68% male). The mean S-Klotho was 860.07 ± 314.15 pg/ml. Table 1 summarizes the target population’s characteristics according to the recommended cut-off of the HEI-2015. Those with higher HEI scores were more likely to be older, female, non-hispanic white, have higher levels of education and income, be physically active and have healthier weight, and be less likely to smoke and drink alcohol (*p* < 0.05). As shown in Table 2, when cardiometabolic biomarkers were compared with HEI-2015 levels, higher HEI-2015 levels were significantly associated with lower levels of glycohemoglobin, glucose, triglycerides, uric acid, and CRP at baseline (*p* < 0.05).

**TABLE 1:** Baseline characteristics of participants.

Characteristic	Healthy Eating Index-2015				*P*-value
	Q1(lowest) ≤ 60	Q2 > 60, ≤ 70	Q3 > 70, ≤ 80	Q4(highest) > 80	
*N* (%)	5859	1581	765	251	
Age (years)	56.76 ± 10.98	58.49 ± 10.77	59.19 ± 10.93	59.98 ± 10.32	<0.001
Sex (%)					<0.001
Female	2757 (47.06%)	837 (52.94%)	427 (55.82%)	139 (55.38%)	
Male	3102 (52.94%)	744 (47.06%)	338 (44.18%)	112 (44.62%)	
Race/Ethnicity (%)					<0.001
Mexican American	894 (15.26%)	210 (13.28%)	83 (10.85%)	23 (9.16%)	
Other Hispanic	581 (9.92%)	216 (13.66%)	87 (11.37%)	31 (12.35%)	
Non-Hispanic white	2538 (43.32%)	655 (41.43%)	358 (46.80%)	116 (46.22%)	
Non-Hispanic black	1375 (23.47%)	280 (17.71%)	127 (16.60%)	42 (16.73%)	
Other race/Ethnicity	471 (8.04%)	220 (13.92%)	110 (14.38%)	39 (15.54%)	
Education (%)					<0.001
Less than 9th grade	748 (12.77%)	173 (10.94%)	71 (9.28%)	17 (6.77%)	
High school or equivalent	2287 (39.03%)	442 (27.96%)	198 (25.88%)	50 (19.92%)	
College or above	2824 (48.20%)	966 (61.10%)	496 (64.84%)	184 (73.31%)	
Smoking status (%)					<0.001
Never	2850 (48.64%)	914 (57.81%)	487 (63.66%)	162 (64.54%)	
Former	1609 (27.46%)	496 (31.37%)	226 (29.54%)	73 (29.08%)	
Now	1397 (23.84%)	171 (10.82%)	52 (6.80%)	15 (5.98%)	
Not recorded	3 (0.05%)	0 (0.00%)	0 (0.00%)	1 (0.40%)	
Drinking status (%)					<0.001
Never	649 (11.08%)	224 (14.17%)	109 (14.25%)	42 (16.73%)	
Former	1212 (20.69%)	261 (16.51%)	117 (15.29%)	30 (11.95%)	
Mild	1878 (32.05%)	613 (38.77%)	339 (44.31%)	129 (51.39%)	
Moderate	815 (13.91%)	196 (12.40%)	114 (14.90%)	32 (12.75%)	
Heavy	992 (16.93%)	199 (12.59%)	46 (6.01%)	10 (3.98%)	
Not recorded	313 (5.34%)	88 (5.57%)	40 (5.23%)	8 (3.19%)	
Family poverty income ratio (%)					<0.001
<1.3	1701 (29.03%)	328 (20.75%)	117 (15.29%)	47 (18.73%)	
1.3–3.5	1992 (34.00%)	471 (29.79%)	226 (29.54%)	68 (27.09%)	
>3.5	1727 (29.48%)	628 (39.72%)	365 (47.71%)	119 (47.41%)	
Not recorded	439 (7.49%)	154 (9.74%)	57 (7.45%)	17 (6.77%)	
Systolic blood pressure(mmHg)	125.21 ± 17.96	123.72 ± 16.86	122.69 ± 17.22	123.24 ± 16.77	<0.001
Diastolic blood pressure(mmHg)	71.00 ± 10.75	70.39 ± 10.50	69.44 ± 10.54	70.41 ± 9.93	<0.001
Body mass index (kg/m^2^)	28.33 ± 6.31	27.80 ± 5.84	26.91 ± 5.19	26.06 ± 5.02	<0.001
Waist circumference (cm)	98.13 ± 14.58	96.54 ± 13.78	94.06 ± 12.40	92.32 ± 12.06	<0.001
Physical activity (MET-min per week)					<0.001
<600	940 (16.04%)	256 (16.19%)	118 (15.42%)	42 (16.73%)	
600–1200	638 (10.89%)	222 (14.04%)	121 (15.82%)	37 (14.74%)	
>1200	2533 (43.23%)	753 (47.63%)	395 (51.63%)	146 (58.17%)	
Not recorded	1748 (29.83%)	350 (22.14%)	131 (17.12%)	26 (10.36%)	
Chronic kidney disease (%)					0.010
Yes	991 (16.91%)	255 (16.13%)	103 (13.46%)	27 (10.76%)	
No	4815 (82.18%)	1318 (83.36%)	656 (85.75%)	224 (89.24%)	
Not recorded	53 (0.90%)	8 (0.51%)	6 (0.78%)	0 (0.00%)	
Energy intake (kcal/day)	2079.58 ± 966.08	1969.12 ± 807.03	1858.11 ± 720.29	1893.81 ± 784.95	<0.001
S-Klotho (pg/ml)	851.64 ± 312.30	878.35 ± 329.71	879.80 ± 312.65	903.58 ± 332.19	<0.001

Data are presented as mean ± SD or *n* (%).

**TABLE 2:** Baseline levels of cardiometabolic markers according to participants.

Characteristic	Healthy Eating Index-2015				*P*-value
	Q1(lowest) ≤ 60	Q2 > 60, ≤ 70	Q3 > 70, ≤ 80	Q4(highest) > 80	
Glycohemoglobin (%)	5.75 ± 0.96	5.71 ± 0.82	5.68 ± 0.83	5.64 ± 0.95	0.043
Glucose, refrigerated serum (mg/dL)	100.64 ± 33.69	99.06 ± 26.98	99.20 ± 30.96	99.65 ± 32.91	<0.001
Cholesterol (mg/dL)	199.15 ± 41.77	198.34 ± 40.72	199.09 ± 39.69	195.73 ± 40.95	0.573
HDL-cholesterol (mg/dL)	57.58 ± 16.22	60.36 ± 17.42	61.12 ± 16.39	61.82 ± 18.25	<0.001
LDL-cholesterol (mg/dL)	118.42 ± 35.91	116.41 ± 33.00	115.61 ± 33.44	113.93 ± 34.37	0.172
Triglycerides (mg/dL)	132.40 ± 92.52	126.69 ± 108.56	120.74 ± 71.79	120.53 ± 75.50	0.001
eGFR (mL/min/1.73 m^2^)	88.01 ± 19.83	87.39 ± 18.55	86.34 ± 18.56	87.73 ± 16.78	0.010
Uric acid (mg/dL)	5.39 ± 1.40	5.34 ± 1.36	5.22 ± 1.33	5.05 ± 1.25	<0.001
C-reactive protein (mg/dL)	0.43 ± 0.87	0.39 ± 0.96	0.31 ± 0.83	0.32 ± 0.48	<0.001

Data are presented as mean ± SD or *n* (%).

### 3.1. Relationships between Healthy Eating Index-2015 and S-Klotho

We designed three multivariate regression models to investigate the relationship between HEI-2015 and S-Klotho. The results of the multivariate regression analyses are presented in Table 3. After multivariate adjustment including age, gender, race/ethnicity, education level, income to poverty ratio, physical activity, energy intake, chronic kidney disease, smoking and drinking habits (Model II),the multivariate-adjusted β and 95% confidence intervals (CIs) from lowest to highest HEI-2015 categories (≤60, 60–70, 70–80, and >80.00) were 1.00 (reference), 23.63 (5.13, 42.12), 23.27 (−1.58, 48.13), and 44.72 (3.59, 85.85), respectively.

**TABLE 3:** Relationship between Healthy Eating Index (HEI)-2015 and S-Klotho.

	Crude model		Model I		Model II	
	β (95% CI)	*P*-value	β (95% CI)	*P*-value	β (95% CI)	*P*-value
HEI-2015	0.93 (0.44, 1.42)	0.0002	1.09 (0.60, 1.58)	<0.0001	0.82 (0.30, 1.35)	0.0023
**HEI-2015 categories**						
≤60	Reference		Reference		Reference	
60–70	26.72 (9.15, 44.28)	0.0029	29.07 (11.59, 46.56)	0.0011	23.63 (5.13, 42.12)	0.0123
70–80	28.16 (4.33, 51.99)	0.0206	32.62 (8.94, 56.30)	0.0070	23.27 (−1.58, 48.13)	0.0665
>80	51.94 (11.98, 91.89)	0.0109	57.94 (18.37, 97.52)	0.0041	44.72 (3.59, 85.85)	0.0331
P for trend	<0.001		<0.001		0.002	

Crude model: no covariates were adjusted. Model I: age, gender, race/ethnicity were adjusted. Model II: age, gender, race/ethnicity, education level, income to poverty ratio, physical activity, chronic kidney disease, smoking and drinking habits were adjusted.

### 3.2. The detection of non-linear relationships

**FIGURE 1:**
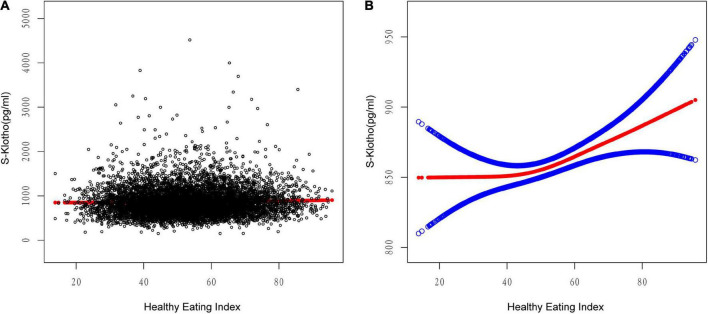
Relationship between HEI-2015 and S-Klotho. **(A)** Each black dot represents A sample. **(B)** Solid lines represent smooth curve fitting between variables. The blue dashed line represents the fit’s 95% confidence interval. They controlled for calorie consumption, age, gender, race/ethnicity, education level, income to poverty ratio, physical activity, energy intake, chronic kidney disease, and smoking and drinking habits.

A scatterplot present the relationship between HEI-2015 and S-Klotho concentrations (Figure 1A). Because HEI-2015 was continuous variable, the analyses of non-linear relationship are necessary. By generalized additive models and smoothed curve fitting and adjusted for potential confounders, we discovered the J-shaped associations between HEI-2015 on S-Klotho in the present study (Figure 1B). Based on this relationship, we do an two-piece-wise regression model analysis and find that it is better than the linear model to explain the relationship using this non-linear model (P for log-likelihood ratio < 0.05), (Table 4). We found the turning point of HEI-2015 was 45.15. When the range of HEI-2015 was from 0 to 45.15, the relationship between HEI and S-Klotho was not significant (β = −0.87, 95% CI: −2.47, 0.73, *P* = 0.2858). However, when the range of HEI-2015 was from 45.15 to 100, HEI-2015 increased by 1 unit, the S-Klotho increased by 1.30 pg/ml (β = 1.30, 95% CI: 0.55, 2.05, *P* = 0.0007).

**TABLE 4:** Treshold effect analysis of Healthy Eating Index (HEI)-2015 on S-Klotho using the two-piecewise linear regression model.

	Adjusted β (95% CI)	*P*-value
Fitting by the standard linear model	0.74 (0.21, 1.27)	0.0067
Fitting by the two-piecewise linear model		
Inflection point	45.15	
HEI-2015 < 45.15	−0.87 (−2.47, 0.73)	0.2858
HEI-2015 > 45.15	1.30 (0.55, 2.05)	0.0007
Log likelihood ratio	0.036	

Age, gender, race/ethnicity, education level, income to poverty ratio, physical activity, body mass index (smooth), chronic kidney disease, smoking and drinking habits were adjusted.

### 3.3. Stratified analyses

**FIGURE 2:**
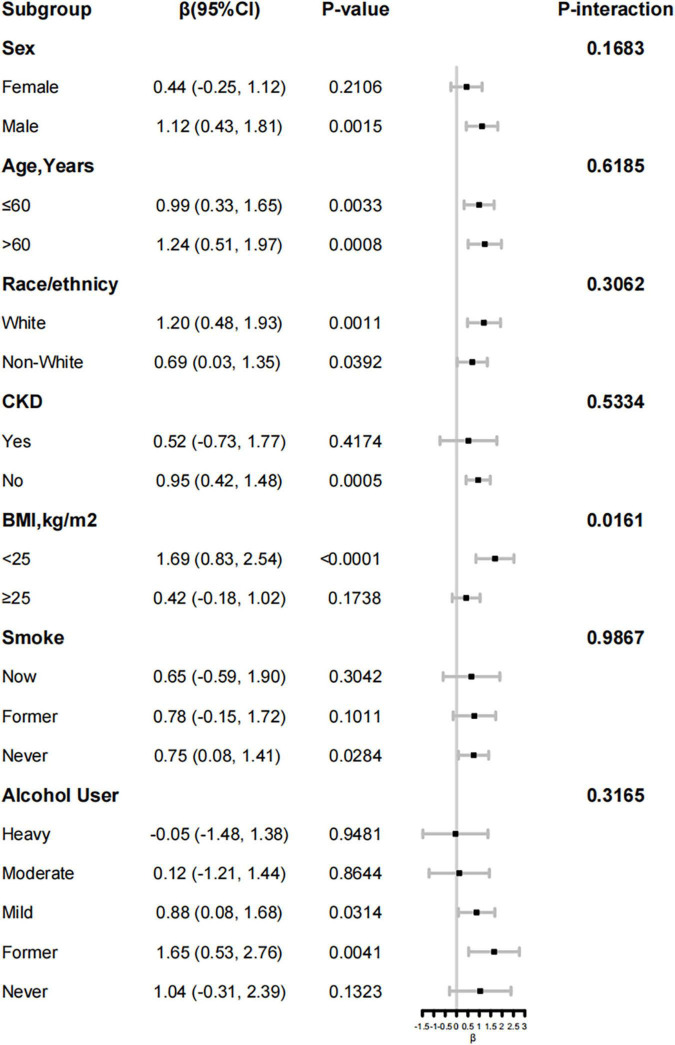
Forest plots of stratified analyses of Healthy Eating Index (HEI)-2015 and S-Klotho.

The advantage of higher HEI on S-Klotho was similar across a wide range of subgroups stratified by age, sex, race, chronic kidney disease, smoking and drinking behaviors (Figure 2). There was a significant interaction between HEI-2015 and S-Klotho stratified by body mass index (P -interaction = 0.0161), such a trend is more common in people of normal body mass index (β = 1.69 95% 0.83, 2.54, *P* < 0.0001). When we limit the analysis to BMI < 25 kg/m^2^ individuals (Table 5), the multivariate-adjusted β and 95% confidence intervals (CIs) from lowest to highest HEI-2015 categories (≤60, 60–70, 70–80, and >80.00) were 1.00 (reference), 32.97 (0.26, 65.68), 70.45 (27.72, 113.18), 81.21 (17.30, 145.12); P for trend < 0.001, respectively.

**TABLE 5:** Relationship between Healthy Eating Index (HEI)-2015 and S-Klotho stratified by BMI.

	Crude model		Model I		Model II	
	β (95% CI)	*P*-value	β (95% CI)	*P*-value	β (95% CI)	*P*-value
**Stratified by BMI**						
**BMI < 25 kg/m^2^**						
HEI-2015	1.69 (0.83, 2.54)	0.0001	1.88 (1.02, 2.75)	<0.0001	1.42 (0.46, 2.38)	0.0037
**HEI-2015 categories**						
≤60	Reference		Reference		Reference	
60–70	37.62 (6.78, 68.47)	0.0169	43.05 (12.28, 73.82)	0.0061	32.97 (0.26, 65.68)	0.0483
70–80	71.50 (31.40, 111.60)	0.0005	74.70 (34.62, 114.77)	0.0003	70.45 (27.72, 113.18)	0.0012
>80	97.74 (36.14, 159.34)	0.0019	105.31 (44.26, 166.35)	0.0007	81.21 (17.30, 145.12)	0.0128
P for trend	<0.001		<0.001		<0.001	
**BMI ≥ 25 kg/m^2^**						
HEI-2015	0.42 (−0.18, 1.02)	0.1738	0.59 (−0.01, 1.19)	0.0555	0.39 (−0.25, 1.04)	0.2296
**HEI-2015 categories**						
≤60	Reference		Reference		Reference	
60–70	21.23 (−0.27, 42.72)	0.0529	22.83 (1.44, 44.21)	0.0364	18.51 (−4.12, 41.13)	0.1089
70–80	−0.63 (−30.65, 29.39)	0.9672	5.83 (−23.95, 35.62)	0.7010	−6.74 (−37.87, 24.40)	0.6715
>80	11.75 (−41.98, 65.47)	0.6683	19.08 (−34.18, 72.34)	0.4826	15.95 (−39.56, 71.46)	0.5734
P for trend	0.353		0.163		0.557	

Crude model: no covariates were adjusted. Model I: age, gender, race/ethnicity were adjusted. Model II: age, gender, race/ethnicity, education level, income to poverty ratio, physical activity, chronic kidney disease, smoking and drinking habits were adjusted.

## 4. Discussion

To the best of our knowledge, this is the first cross-sectional study with a relatively larger sample size to assess the relationship between HEI-2015 and S-Klotho levels. In the representative study of our participants, there may be a positive correlation between HEI-2015 and S-Klotho levels, and it appears that the J-shaped or dose-response relationship is more appropriate to explain it. After controlling the covariates, no correlation was observed at low HEI-2015 levels (below the inflection point); however, high HEI-2015 levels (above the inflection point) could result in higher plasma levels of S-Klotho. Furthermore, the stratified analysis revealed that the observed positive trend of correlation between HEI-2015 and S-Klotho concentrations differed significantly by body mass index, and it was more pronounced in those with normal body mass index.

Higher HEI scores are associated with a lower risk of chronic diseases; a 25% higher diet score can reduce the risk of cardiovascular disease by 38%, breast cancer by 46%, and all-cause mortality by 12–28% (23–26). Moreover, HEI is designed based on the US dietary guidelines and measures the extent to which individuals comply with these guidelines. The United States has the lowest adherence to the Mediterranean diet (27). The Mediterranean diet may not be a viable choice for most American adults due to differences in dietary culture and availability; thus, this study employed the HEI. Data collection in NHANES takes place on all days of the year, and the likelihood of day-to-day information bias is minimal.

Compared to the study of single foods or nutrients, people’s daily diet is a mixture of multiple foods and nutrients. Complete diet patterns, including the HEI, the Inflammatory Diet Index (DII), the Mediterranean Diet (MedDiet), and the Vegan Diet, have received much attention in recent years. Fortunately, NHANES has provided sufficient data to further explore this interesting issue. Although these dietary quality measures are not entirely consistent, previous studies have demonstrated similar associations between different dietary qualities and S-Klotho. For example, both Chi-Chen and our previous NHANES studies imply that an inflammatory diet may lower S-Klotho levels (28, 29). Shou-En found a positive association between Mediterranean adherence diet score (MDS) and S-Klotho levels based on NHANES. However, the other three dietary patterns, low-carbohydrate diet score, low-fat diet, and low-carbohydrate diet, showed no association (30). Fasoli, on the other hand, found a negative correlation between MDS and S-Klotho levels in sedentary middle-aged adults based on a secondary analysis of participants in the FIT-AGING program (31). The discrepancy in sample size and target population could explain the disparity in outcomes.

According to DGA, the HEI score is a well-validated indicator of dietary quality, and adherence to a high-quality diet is significantly negatively correlated with various biomarkers of chronic low-grade inflammation, which is characterized by a decrease in free radicals and an accumulation of other oxides, resulting in an imbalance in the antioxidant mechanism (32, 33). Many inflammatory factors, including IL-6, IL-1β, TNF-α, and TGF-β, are involved in this chronic low-grade inflammation process, blocking the transcription of the Klotho gene, and reducing the level of S-Klotho (34–36). Furthermore, the Klotho protein is an important component of the endocrine fibroblast growth factor (FGF) receptor complex, and the formation of the Klotho/FGF23/FGFR complex is essential for the transduction of FGF signaling. This signaling can help stimulate insulin sensitivity and glucose metabolism by activating the insulin/IGF1/IGF1R ligand/receptor complex, thereby reducing body weight (37). This finding is consistent with the trends observed by our stratification-based analysis.

The current research has some limitations. First of all, it has a horizontal design that excludes the construction of causality. Second, we do not know whether these results can be extended to young adults. Larger prospective studies investigating the association between HEI and S-Klotho are needed to determine whether these results are reproducible in the population. Third, it is critical to consider the challenges associated with proper dietary assessment; dietary data are subject to recall bias and these challenges may be exaggerated or misclassified. Finally, unknown or unmeasured confounding variables may affect our results.

## 5. Conclusion

There is a dose-response relationship between the HEI-2015 and S-Klotho in the middle-to-older aged adults. This relationship suggests that adherence to healthy dietary patterns may benefit the prevention of aging and health maintenance. The underlying mechanisms require further investigation.

## Data availability statement

Publicly available datasets were analyzed in this study. This data can be found here: https://wwwn.cdc.gov/nchs/nhanes/search/default.aspx.

## Ethics statement

The studies involving human participants were reviewed and approved by the National Center for Health Statistics, Research Ethics Review Board (ERB). The patients/participants provided their written informed consent to participate in this study.

## Author contributions

T-CM designed the current research and made data analysis. C-XW, Z-ZL, and JZ made equal contributions to the writing of the manuscript. FG critically revised and edited the manuscript for significant intellectual content. All authors reviewed and approved the final manuscript.

## Footnotes

1
http://www.r-project.org

2
www.empowerstats.com
